# Cholesterol Granuloma in the Maxillary Sinus: Are Endodontically Treated Teeth Involved in Its Etiopathogenesis?

**DOI:** 10.1155/2017/5249161

**Published:** 2017-10-19

**Authors:** Silas Antonio Juvencio de Freitas Filho, Gilberto Gallo Esteves, Denise Tostes Oliveira

**Affiliations:** ^1^Department of Surgery, Stomatology, Pathology and Radiology, Area of Pathology, Bauru School of Dentistry, University of São Paulo, Bauru, SP, Brazil; ^2^Private Practice, Marília, SP, Brazil

## Abstract

Cholesterol granuloma (CG) is a tissue reaction in response to the accumulation of cholesterol crystals rarely found in the maxillary sinus. The etiopathogenesis of maxillary sinus CG remains unclear. We reviewed the literature and added two new reports of cholesterol granuloma in maxillary sinus related to endodontically treated maxillary posterior teeth. The first report refers to a 45-year-old woman diagnosed with rhinitis, who was submitted to endodontic retreatment of maxillary molar, and subsequently showed maxillary sinus opacity with cystic appearance. The second case describes a young adult woman, who presented a cystic mass in maxillary sinus after endodontic treatment, in close association with the apex of the maxillary right second premolar. Both patients were treated by a classic Caldwell-Luc surgery and the microscopic analyses revealed maxillary sinus CG. In the following, the authors discuss the probable involvement of endodontically treated maxillary posterior teeth in the etiopathogenesis of maxillary sinus CG.

## 1. Introduction

Cholesterol granuloma (CG) is considered rare in maxillary sinus and approximately 50 cases were reported in English literature, with 12 of them being described from 2005 to 2016 [1–10]. The clinical features of CG in the maxillary sinus are nonspecific mimicking other cystic or inflammatory diseases [3]. It is often associated with a history of rhinitis, sinusitis, trauma, and paranasal sinus surgery [6, 9, 10] and can be accompanied by symptoms, such as facial pain, headache, otalgia, rhinorrhea, and nasal obstruction, commonly showing a cystic appearance and sinus opacification in radiological examinations [2, 3].

Diagnosis of maxillary sinus CG is based on microscopic analysis of a foreign body reaction characterized by foreign body giant cells and longitudinal cholesterol clefts, granulocytes, foam cells, and macrophages filled with hemosiderin [2, 3]. Completing the findings, it is possible to observe fibrin deposition and local bleeding [3, 7].

Here we intend to report two cases of cholesterol granuloma in the maxillary sinus and to discuss the involvement of endodontically treated maxillary posterior teeth in their probable pathogenesis.

## 2. Case Report

### 2.1. Case 1

**Figure 1:**
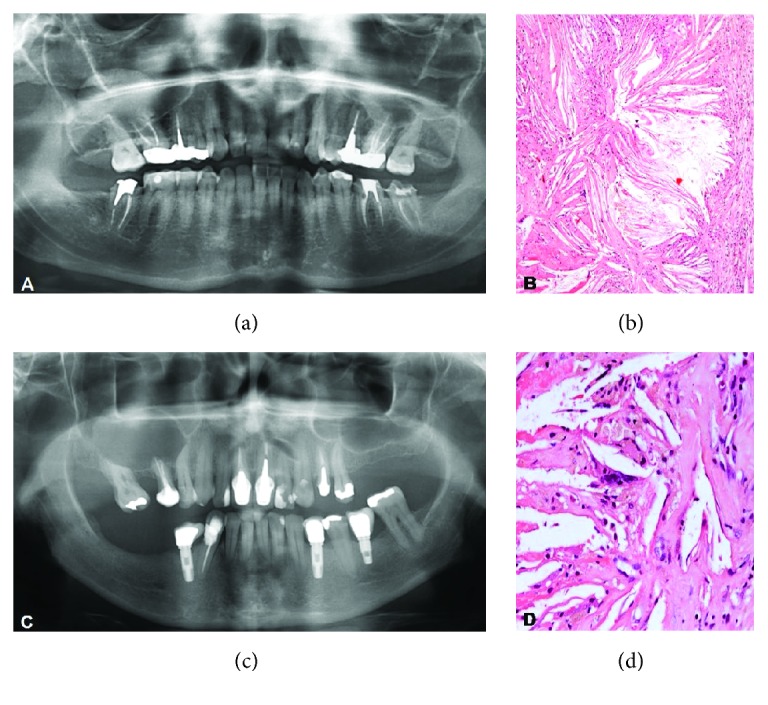
(a) Opacity and cystic appearance in the right maxillary sinus associated with the roots of the maxillary right first molar; (b) cholesterol granuloma characterized by numerous longitudinal clefts permeated by irregular eosinophilic material surrounded by foreign body giant cells and macrophages (hematoxylin-eosin stain, ×100); (c) cystic mass in maxillary sinus in close association with the apex of the maxillary right second premolar; (d) foreign body reaction with accumulation of cholesterol clefts involving macrophages and foreign body giant cells (hematoxylin-eosin stain, ×400).

A 45-year-old woman was attended in dental clinic, seeking oral rehabilitation. Clinical and radiographic examination revealed that the patient had undergone unsatisfactory endodontic treatment in the maxillary right first primary molar and experienced painful symptomatology in this tooth which was then submitted to endodontic retreatment. After this dental procedure, the patient reported pain, swelling, and nasal congestion, and a medication for rhinitis was prescribed. Radiographic evaluation revealed opacity and cystic appearance in the right maxillary sinus associated with the roots of the maxillary right first molar (Figure 1(a)). An excisional biopsy was performed in the maxillary sinus for histopathological analysis. Microscopic examination showed the maxillary sinus mucosa composed of ciliated pseudostratified columnar epithelium. In the submucosa, numerous longitudinal clefts were observed after dissolution of the cholesterol crystals; these were permeated by irregular eosinophilic material and surrounded by foreign body giant cells and macrophages (Figure 1(b)). Furthermore, the presence of edema, fibrin, and diffuse chronic inflammatory infiltrate was found. The final diagnosis was maxillary sinus CG associated with maxillary sinusitis. After surgical excision, the clinical symptoms were no longer reported.

### 2.2. Case 2

A young adult woman was attended in dental clinic, reporting pain in the right facial region. In the clinical and radiographic history, a cystic mass was observed in the right maxillary sinus in contact with root apex of the endodontically treated maxillary right second premolar (Figure 1(c)). The lesion was surgically removed and the histopathological analysis showed subjacent to normal sinus mucosa, the presence of intense mononuclear inflammatory infiltrate, cholesterol clefts surrounded by foreign body giant cells, and foci of hemosiderosis and hemorrhagic areas. Based on the microscopic features, the diagnosis of maxillary sinus CG associated with maxillary sinusitis was established.

## 3. Discussion

Cholesterol granuloma in the maxillary sinus, as reported in our cases, is a histologic finding associated with clinical symptoms of inflammatory diseases such as rhinitis and sinusitis [3, 9]. According to the literature review summarized in the Table 1, few cases of CG have been described in maxillary sinus between 2005 and 2016, and the age of these patients ranged from 22 to 67 years (mean of 44.9 years), with an average follow-up of 13.4 months. Pain and nasal obstruction/congestion were the most common symptoms reported (Table 1), including our case, number one. Golden yellow rhinorrhea is another significant symptom [3], but our patients did not present this specific symptom. Furthermore, opacity of the maxillary sinus was frequently observed by computed tomography or by panoramic radiography [4–7, 9, 10]. Although some authors recommended computed tomography as the most appropriate exam for identifying changes in the maxillary sinus [9, 10], in our cases the radiographic images were sufficient to detect the relationship between tooth roots and the maxillary sinus lesions.

The clinical symptoms of maxillary sinus CG, such as those described above, also found in our patients, were unspecific and mimicked other common inflammatory sinus diseases [3, 5]. The differential diagnosis of CG included chronic allergic sinusitis, mucoceles, pyomucoceles, odontogenic cysts, and neoplasms [9]. Therefore, the final diagnosis of maxillary sinus CG should be established after histopathological analysis. The presence of foreign body reaction with accumulation of cholesterol clefts involving macrophages and foreign body giant cells constituted the main feature (Figure 1(d)). The persistence of these cells may be the major source of cholesterol that is released by the destroyed membranes of these cells in chronic lesions [1, 2, 11].

The pathogenesis of maxillary sinus CG is still uncertain and its occurrence has been reported secondarily to bleeding, inadequate lymphatic drainage, poor ventilation, trauma, surgery, sinusitis, and odontogenic lesions [3, 7, 11, 12]. It has been suggested that inflamed odontogenic lesions could show a foreign body reaction to the cholesterol crystals in their capsule, and as the capsule expanded it may possibly reach the maxillary sinus [7]. In addition, inadequate drainage and hemorrhage into the bone cavity [3], or inflammatory processes causing focal hemorrhage, have been implicated in the development of maxillary sinus CG [1, 12]. In these conditions, the cholesterol crystals arising from the plasma membrane of destroyed red blood cells [6, 9] induced an inflammatory reaction of the foreign body type.

The association of CG with maxillary sinusitis, such as that found in our cases, was a common occurrence [1, 5, 8] and according to meta-analysis of Arias-Irimia et al. [13] the odontogenic conditions were etiologic factors involved in 47.68% of the cases of maxillary sinusitis. The interesting feature in our cases was the occurrence of maxillary sinus CG in close association with endodontically treated maxillary posterior teeth. The development of maxillary sinusitis, including the occurrence of CG, could be attributed to hemorrhage and inflammatory processes in maxillary sinus mucosa after instrumentation or endodontic obturation [13]. The anatomical relationship between apices of posterosuperior teeth and the floor of the maxillary sinus may explain some sinus diseases [14, 15]. It has been established that iatrogenia is an etiologic factor in the development of odontogenic maxillary sinusitis associated with the extrusion of endodontic obturation materials [13]. Our cases presented and reinforced the importance of careful investigation of the relationship between roots of maxillary posterior teeth and inflammatory processes of the maxillary sinus suggesting that endodontic material extruded into the antrum could be responsible for foreign body reactions and the maintenance of chronic inflammatory processes in this location.

Furthermore, the accumulation of cholesterol crystals associated with macrophages and giant cells could induce delayed tissue repair after conventional endodontic treatment because the macrophages and giant cells are the main sources of inflammatory mediators contributing to the maintenance of chronic inflammation and clinical symptoms [16].

The most appropriate treatment of CG in the maxillary sinus was based on surgery, commonly by means of a classic Caldwell-Luc operation [3]. Endoscopic sinus surgery is another possible approach that could be clinically used [3, 10]. Our cases were treated by the Caldwell-Luc approach and no problems were reported.

In summary, we presented an updated literature review and two new reports of maxillary sinus CG in close association with endodontically treated maxillary posterior teeth, reinforcing the probable involvement of the presence of endodontic material in the etiopathogenesis of chronic sinusitis, particularly in a foreign body reaction induced by cholesterol crystals. Furthermore, in cases of maxillary sinus lesions, teeth and dental procedures should be evaluated.

## Figures and Tables

**Table 1:** Previously reported cases of cholesterol granuloma in maxillary sinus with clinical-radiographic findings (2005–2016).

Authors (country)	Age	Gender	Side	Symptoms/signs	Radiographic characteristics	Tooth vitality	Treatment	Follow-up (without recurrence)
Bella et al., 2005 (Hungary) [1]	63	F	R	Pain, purulent discharge, obstruction of right nasal cavity	Homogeneous shadow^*∗*^, mass destroying the lateral nasal cavity wall [2]	NI	Surgical excision (CL)	12 months
Chao, 2006 (Taiwan) [3]	42 56	M F	B R	Blood-tinged sputum, an episode of left golden yellow nasal rhinorrhea Blood-tinged sputum, foul smelling	Cystic lesions^NI^ Cystic lesion^NI^	NI NI	Endoscopic sinus surgery Endoscopic sinus surgery	14 months 2 months
Ko et al., 2006 (Taiwan) [4]	34	F	L	Nasal obstruction, facial pain	Opacity, STM^†^	NI	Functional endoscopic sinus approach	36 months
Marina and Gendeh, 2006 (Malaysia) [5]	26	M	B	Headache, bilateral interchangeable nasal blockage, pain	Cystic lesion^†^	NI	Sublabial antrostomies and surgical excision	12 months
Ramani et al., 2006 (India) [6]	42	M	L	Headaches, pain, nasal congestion	Opacity^*∗*^, mucosal thickening^†^	NI	Surgical excision (CL)	NI
Almada et al., 2008 (Brazil) [7]	22	M	R	A tender painful swelling on the right maxilla, fistula, pain associated with nasal obstruction	Opacity with bone expansion and destruction^†^	Yes	NI^‡^	NI
Astarci et al., 2008 (Turkey) [8]^§^	33	M	B	Difficulty in nasal breathing Sleeping with open mouth	STM^†^	NI	Endoscopic approach	NI
Alzahrani et al., 2010 (France) [9]	37	M	R	Acute febrile sinusitis	Opacity^*∗*,†^	NI	Functional endoscopic sinus approach with marsupialization	12 months
Karaky et al., 2010 (Jordan) [2]	60	F	R	Upper posterior alveolar ridge resorbed	No radiographic changes	Teeth absent	Enucleation, curettage	6 months
Alkan et al., 2014 (Turkey) [10]	57 67	M F	R L	Asymptomatic, history of trauma Painful expansion	Radiolucent with focal radiopaque appearance^*∗*^, opacity with bone destruction^†^ Radiolucent^*∗*^	NI NI	Surgical excision Surgical excision	NSR NI
Our cases, 2017 (Brazil)	45	F	R	Pain, swelling, nasal congestion	Opacity, cystic appearance^*∗*^	Devitalized	Surgical excision (CL)	76 months
	NI	F	R	Facial pain	Cystic lesion^*∗*^	Devitalized	Surgical excision (CL)	NI

M: male; F: female; R: right; L: left; B: bilateral; NI: not informed; STM: soft tissue mass; CL: Caldwell-Luc operation; NSR: no signs of recurrence; ^*∗*^panoramic radiographic; ^†^computed tomography; ^‡^performed only incisional biopsy; ^§^patient also presented CG in the ethmoid sinus.
